# EV-transferred miR-7111-3p inhibits malignant phenotypes and tumor growth in nasopharyngeal carcinoma via targeting APP-regulated epithelial-mesenchymal transition

**DOI:** 10.1186/s12967-026-08674-1

**Published:** 2026-07-30

**Authors:** Hejing Huang, Zhehao Xiao, Weiling Chen, Zhao Li, Xuxia Chen, Xiaohui Yang, Xiaojun Chen, Ziman Luo, Huining Chen, Song Qu

**Affiliations:** 1 Guangxi Medical University Cancer Hospital, Nanning, Guangxi China; 2 Guangxi Medical University, Nanning, Guangxi China; 3 Key Laboratory of Early Prevention and Treatment for Regional High Frequency Tumor (Gaungxi Medical University), Ministry of Education, Nanning, Guangxi China

**Keywords:** Nasopharyngeal carcinoma, miR-7111-3p, Extracellular vesicles, Invasion and metastasis

## Abstract

**Background and purpose:**

Extracellular vesicle (EV)-mediated transfer of microRNAs has emerged as a critical mechanism driving tumor progression, yet its functional relevance in nasopharyngeal carcinoma (NPC) remains poorly defined. This study sought to elucidate the functional role and molecular mechanism of EV-associated miR-7111-3p in NPC progression.

**Methods:**

miR-7111-3p expression was measured in serum samples from NPC patients and in NPC cell lines. Functional effects were evaluated through gain- and loss-of-function assays in vitro and in xenograft models. Direct targeting of amyloid precursor protein (APP) by miR-7111-3p was validated using dual-luciferase reporter assays. EV-mediated transfer was assessed by EV isolation, characterization, and co-culture experiments.

**Results:**

miR-7111-3p was significantly downregulated in NPC serum and cell lines. Its overexpression markedly suppressed NPC cell proliferation, migration, invasion, and epithelial-mesenchymal transition (EMT), whereas its knockdown exerted the opposite effects. APP was identified as a direct target of miR-7111-3p, and APP overexpression reversed the inhibitory effects induced by miR-7111-3p. EVs derived from miR-7111-3p-deficient NPC cells promoted APP expression, proliferation and migration in recipient NP69 cells, whereas inhibition of EV secretion attenuated these effects. In vivo, miR-7111-3p overexpression reduced xenograft tumor growth and downregulated APP expression.

**Conclusion:**

These findings identify a novel EV-miR-7111-3p-APP signaling axis that regulates EMT, malignant phenotypes and tumor growth in NPC, highlighting its potential as a therapeutic target.

**Supplementary Information:**

The online version contains supplementary material available at https://doi.org/10.1186/s12967-026-08674-1.

## Introduction

Nasopharyngeal carcinoma (NPC) is a highly invasive epithelial malignancy characterized by a distinct geographic distribution, with the highest incidence reported in Southern China and Southeast Asia [1, 2]. Despite substantial advances in radiotherapy and chemoradiotherapy, locoregional recurrence and distant metastasis remain major causes of treatment failure and cancer-related mortality, particularly in patients with advanced disease [3, 4]. Therefore, elucidating the molecular mechanisms underlying NPC invasion and metastasis is crucial for the development of effective therapeutic strategies and prognostic biomarkers.

Epithelial-mesenchymal transition (EMT) is a fundamental biological process through which epithelial tumor cells acquire mesenchymal traits, including enhanced migratory and invasive capacities. During EMT, epithelial cells lose cell-cell adhesion and polarity while gaining mesenchymal characteristics, thereby facilitating tumor dissemination and metastatic colonization [5, 6]. Accordingly, elucidating of EMT regulatory mechanisms in NPC is of considerable scientific and clinical significance for understanding disease progression and improving therapeutic strategies.

In recent years, microRNAs (miRNAs), a class of single-stranded non-coding RNAs approximately 18–25 nucleotides in length, have emerged as important regulators in cancer biology and targeted therapy. miRNAs function primarily by binding imperfectly to the 3’-untranslated region (3’UTR) of target mRNAs, leading to mRNA degradation or translational repression, thereby modulating gene expression at the post-transcriptional level. Dysregulation of miRNA expression is closely associated with the initiation and progression of various malignancies [7, 8]. However, the biological function and regulatory mechanism of miR-7111-3p in NPC have not yet been investigated.

Amyloid precursor protein (APP) is a transmembrane protein involved in diverse cellular processes, including axonal growth, cell proliferation, adhesion, and migration [9]. Previous studies have shown that APP knockdown suppresses invasion and migration in NPC SUNE-1 cells, reduced metastasis-associated gene expression, and increases metastasis suppressor genes [10]. In addition, APP has been suggested to promote EMT via activation of the MAPK signaling pathway, thereby enhancing tumor cell migration and invasion in human breast cancer [11]. These findings indicate that APP plays a critical role in tumor invasion and metastasis.

Tumor-derived extracellular vesicles (EVs) are nano-sized membrane-bound vesicles actively secreted by tumor cells, including exosomes (30–150 nm), microvesicles, and apoptotic bodies. EVs are widely present in biological fluids such as blood and saliva and serve as key mediators of intercellular communication [12]. They carry diverse bioactive molecules, including proteins, nucleic acids (e.g., miRNAs and lncRNAs), and lipids, and play essential roles in tumor microenvironment remodeling, immune evasion, angiogenesis, and metastasis [13]. Recent evidence suggests EVs mediate intercellular transfer of molecular signals, influencing not only neighboring cells but also the formation of pre-metastatic niches that facilitate tumor progression. For instance, N2 tumor-associated neutrophils (N2 TANs) have been reported to deliver miR-4745-5p/3911 via exosomes, thereby downregulating SLIT2 and promoting gastric cancer metastasis [14]. However, whether miR-7111-3p is encapsulated within NPC-derived EVs and participates in EV-mediated signaling remains unclear.

Nevertheless, the role of EV-transferred miRNAs in NPC, particularly in EMT regulation and metastasis, has not been fully elucidated. Our preliminary data indicate that miR-7111-3p is associated with radioresistance and invasive potential in NPC, yet its mechanism of action via EVs remains unknown. In this study, we investigate whether miR-7111-3p is delivered by NPC-derived EVs to recipient cells, where it targets APP and regulates EMT-driven invasion and metastasis. This work aims to provide new insights into EV-mediated intercellular communication in NPC and to identify potential therapeutic targets for advanced disease.

## Methods

### Clinical samples

Peripheral blood samples (5 mL, fasting) were collected from 11 histopathologically confirmed NPC patients and 11 healthy volunteers at the Guangxi Medical University Cancer Hospital between January and September 2025. Written informed consent was obtained from all participants. The study was approved by the Institutional Ethics Committee (KY2024308). Serum was isolated by centrifugation (3,000 rpm, 10 min, 4 °C) and stored at -80 °C until analysis.

### Cell culture and transfection

The normal nasopharyngeal epithelial cell line NP69 and NPC cell lines (5–8 F, 6-10B, HK-1, CNE-2, C666-1) were obtained from the Key Laboratory of Guangxi Medical University. NP69 cells were cultured in Keratinocyte-SFM medium (Gibco, 10744019) supplemented EGF and bovine pituitary extract, while other cell lines were maintained in RPMI-1640(Gibco, 11875093) containing 10% fetal bovine serum (FBS) and 1% penicillin–streptomycin at 37 °C with 5% CO₂.

Lentiviral vectors for miR-7111-3p overexpression (OE) and knockdown (shRNA) were purchased from Genechem. Transfection was performed at 70–80% confluence following the manufacturer’s protocol. Transfection efficiency was validated by reverse transcription quantitative polymerase chain reaction (RT-qPCR) 24–48 h post-transfection. Sequences are listed in Supplementary Table S1.

### EVs isolation and characterization

Cells were cultured in EV-depleted medium for 48 h. Supernatants were sequential centrifugation (300 ×g, 10 min; 2,000 ×g, 10 min; 16,500 ×g, 20 min) at 4 °C to remove cells and debris. The resulting supernatant was ultracentrifuged at 100,000 ×g for 2 h at 4 °C to pellet EVs. EV pellets were resuspended in 100 µL of phosphate-buffered saline (PBS) and stored at -80 °C. EV isolation and characterization were performed in accordance with the MISEV2018/2023 guidelines. EV morphology was observed by transmission electron microscopy (TEM), size distribution was analyzed by nanoparticle tracking analysis (NTA), and markers (HSP70 #66183-1-Ig, Proteintech; TSG101 #67381-1-Ig, Proteintech; CD63 #67605-1-Ig, Proteintech) and negative marker Calnexin (#10427-2-AP, Proteintech) were detected by Western blot [15].

### RT-qPCR

Total RNA was extracted using TRIzol (Takara, 9109). cDNA was synthesized using commercial reverse transcription kits (Sangong, B639252-0100 for mRNA, B532451-0050 for miRNA). RT-qPCR was carried out using 2× SG Fast qPCR Master Mix (B639271-0005) and a miRNA-specific fluorescent quantification kit (B532461-0002). GAPDH and U6 served as internal controls for mRNA and miRNA, respectively. Relative expression levels were calculated using the 2 − ΔΔCt method. Primer sequences are listed in Table 1.

**Table 1:** Gene primer sequences

Primer Name	Primer sequence
miR-7111-3p	F: CGATCCTCTCTTCCCTCCTCCCAG R: TGAAGACACAGCAGATGACAGATATGG
APP	F: AGTGACAATGTGGATTCTGCTGATG R: ACTTCTTCCTCCTCTGCTACTTCTAC
E-cadherin	F: TGCCCAGAAAATGAAAAAGG R: GTGTATGTGGCAATGCGTTC
N-cadherin	F: AGCCAACCTTAACTGAGGAGT R: GGCAAGTTGATTGGAGGGATG
Vimentin	F: GAGAACTTTGCCGTTGAAGC R: GCTTCCTGTAGGTGGCAATC
U6	F: CGCGATATGGTTTTGGCAGG R: TGAAGACACAGCAGATGACAGATATGG
GAPDH	F: CAGGAGGCATTGCTGATGAT R: GAAGGCTGGGGCTCATTT
Cel-miR-39	UCACCGGGUGUAAAUCAGCUUG

### Western blot (WB)

Cells were lysed in RIPA buffer with protease inhibitors. Protein concentration was determined using a BCA assay. Equal protein amounts were separated by 10% SDS-PAGE, transferred to PVDF membranes. Membranes were blocked with 5% skim milk for 90 min, incubated with primary antibodies (anti-APP #R013864, Yamei; anti-N-cadherin #13116S, CST; anti-vimentin #5741S, CST; anti-β-actin #66009-1-Ig, Proteintech) overnight at 4 °C, followed by incubation with secondary antibodies (Goat anti-Rabbit/Mouse IgG(H) #SA5-35571/SA5-35521, Thermo Fisher) for 45 min. Signals were detected using an Odyssey CLx imaging system. The dilution ratios for all antibodies are presented in Supplementary Table S2. Band intensity was quantified using ImageJ software.

### Functional assays

Cell proliferation: For the CCK-8 assay, 5 × 10^3 cells/well were seeded in 96-well plates. 10 µL of CCK-8 reagent was added daily and incubated for 2 h (37 °C, 5% CO2), followed by absorbance measurement at 450 nm. Colony formation: 800 cells/well were seeded in 6-well plates and cultured for 14 days. Colonies were fixed with 4% paraformaldehyde, stained with 1% crystal violet, and counted using Image J.

Cell migration and invasion: Wound healing assays were performed using Ibidi inserts. After creating a scratch, cells were cultured in medium containing 1% FBS, and wound closure was monitored at 0, 12–24 h. Migration area was quantified using ImageJ by measuring wound closure percentage. For Transwell assays, 8 μm pore size chambers (Corning) were used. For migration assays, 5 × 10⁴ cells in serum-free medium were seeded in the upper chamber, and medium containing 20% FBS was placed in the lower chamber. For invasion assays, membranes were pre-coated with Matrigel (diluted 1:8 in serum-free medium). After 24–48 h incubation, non-migrated/invaded cells on the upper surface were removed with a cotton swab. Cells on the lower surface were fixed, stained with crystal violet, and counted in five random fields per well. Quantification of all results from these functional assays was performed using Image J.

### Target gene prediction and dual-luciferase reporter assay

APP was predicted as a potential target of miR-7111-3p using TargetScan, miRWalk, and miRTarBase. Wild-type or mutant APP 3′UTR fragments were cloned into luciferase vectors and co-transfected into HEK-293T cells with miR-7111-3p mimics or controls. Dual-luciferase activity (Firefly/Renilla ratio) was measured 48 h post-transfection [16].

### Dataset analysis

The GSE139164 dataset (7 radiosensitive, 5 radioresistant NPC serum samples) was analyzed for differential gene expression and visualized using volcano plots and heatmaps.

### EV uptake and co-culture

Isolated EVs were labeled with the lipophilic dye DIR (Umibiotech) at 37 °C for 1 h, and excess dye was removed by ultracentrifugation. Labeled EVs were incubated with NP69 cells for 12 h. Cells were then fixed, stained with phalloidin (Abcam) and DAPI (Beyotime), and imaged using confocal microscope.

For co-culture, NP69 cells were seeded in the lower chamber of a Transwell system, while C666-1 cells (Vector, miR-7111-3p OE, or miR-7111-3p OE treated with the EV secretion inhibitor GW4869 [10 µmol/L]) were placed in the upper chamber. After 48 h of co-culture, NP69 cells were harvested for analyses.

### Animal experiments

BALB/c nude mice (*n* = 10) were randomly assigned to two groups and subcutaneously injected with C666-1 cells(1 × 10⁶) expressing vector or miR-7111-3p OE. Tumor volume was calculated using the formula: volume = (length × width²)/2. Investigators were blinded to group allocation during outcome assessment. After 4 weeks, mice were euthanized, tumors were excised, weighed, and processed for RNA/protein extraction, immunohistochemistry (IHC), and fluorescence in situ hybridization (FISH). IHC staining intensity was quantified using semi-quantitative scoring.

### miRNA fluorescence in situ hybridization (FISH)

We performed the FISH assay using a fluorescent probe for miR-7111-3p that was specifically designed and synthesized by Servicebio (Wuhan, China). Hybridization was carried out with the fluorescently labeled single-strand probes, following the manufacturer’s instructions of the SweAMI-FISH kit. The detailed protocol was adapted from a previously published method [17].

### Statistical analysis

Data are presented as mean ± standard deviation (SD). Unpaired two-tailed Student’s t-test was used for comparisons between two independent groups. One-way ANOVA coupled with Tukey’s post hoc test was applied for statistical comparisons among three or more experimental groups to correct multiple-comparison bias. All statistical analyses were performed using SPSS 26.0 and GraphPad Prism 9.0. A P value < 0.05 was considered statistically significant.

## Results

### Expression characteristics of miR-7111-3p in NPC

**Fig. 1:**
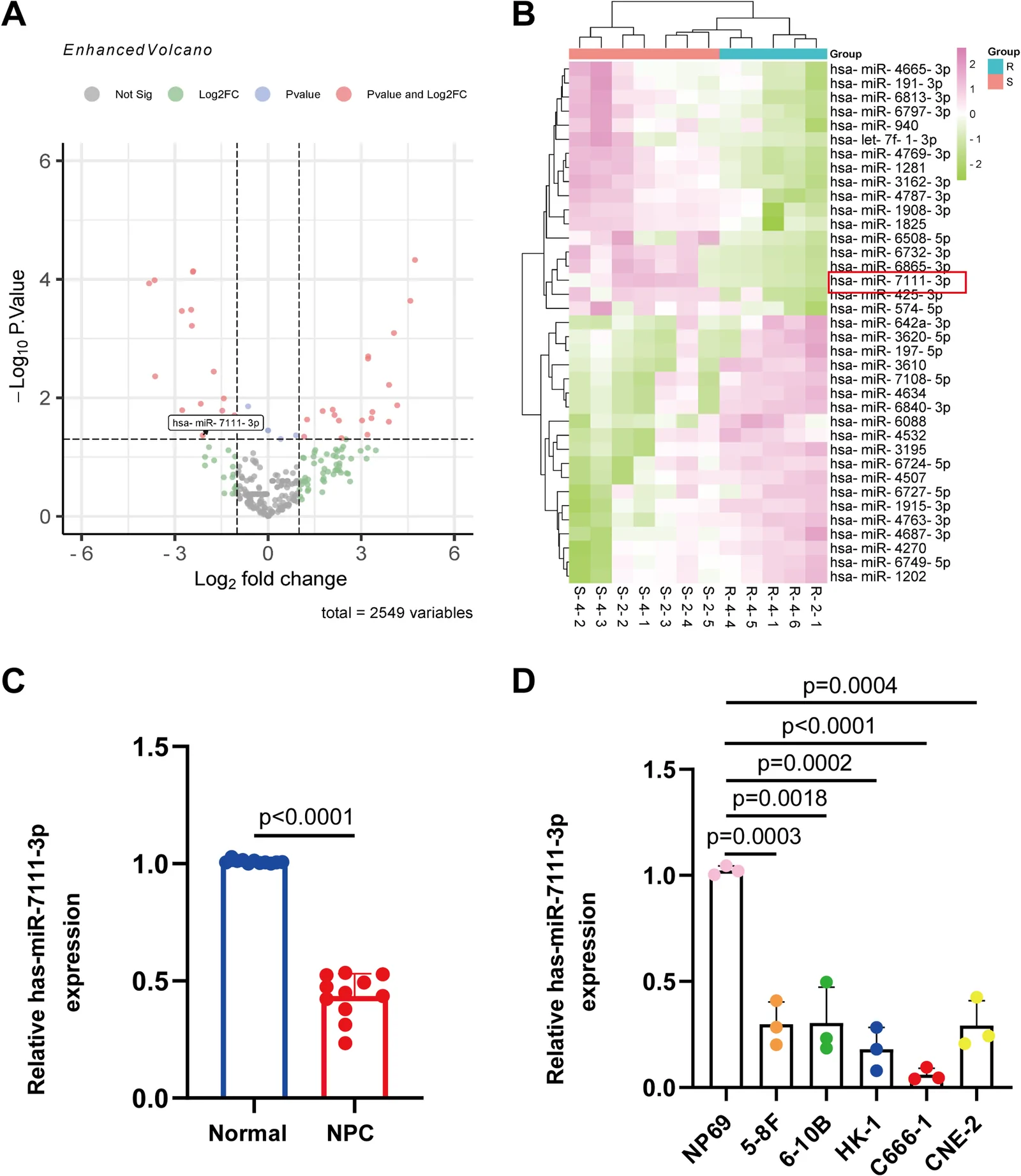
miR-7111-3p expression is decreased in nasopharyngeal carcinoma (NPC). (**A**) Volcano plot showing differentially expressed miRNAs from sequencing of serum samples from radioresistant versus radiosensitive patients. (**B**) Heatmap showing differentially expressed miRNAs from sequencing of serum samples from radioresistant versus radiosensitive patients. (**C**) RT-qPCR analysis of miR-7111-3p expression in serum from healthy donors and NPC patients. (**D**) RT-qPCR analysis of miR-7111-3p expression in normal nasopharyngeal epithelial cells and different NPC cell lines. All cellular experiments were independently repeated for three biological replicates

To investigate the involvement of miR-7111-3p in NPC progression, we first analyzed the GSE139164 dataset. Volcano plot and heatmap analyses revealed that miR-7111-3p was significantly downregulated in serum from radioresistant NPC patients compared with radiosensitive patients (Fig. 1A, B), suggesting an association with aggressive disease phenotypes.

These findings were validated by RT-qPCR in clinical serum samples, where circulating miR-7111-3p levels were significantly reduced in NPC patients compared with healthy controls (*P* < 0.05; Fig. 1C). Consistently, miR-7111-3p expression was markedly decreased in all NPC cell lines (C666-1, HK-1, 5–8 F, 6-10B, and CNE-2) relative to NP69 cells (all *P* < 0.05; Fig. 1D). These results indicate that miR-7111-3p is consistently downregulated in NPC and may function as a tumor-suppressive miRNA.

### Tumor-suppressive effects of miR-7111-3p overexpression

**Fig. 2:**
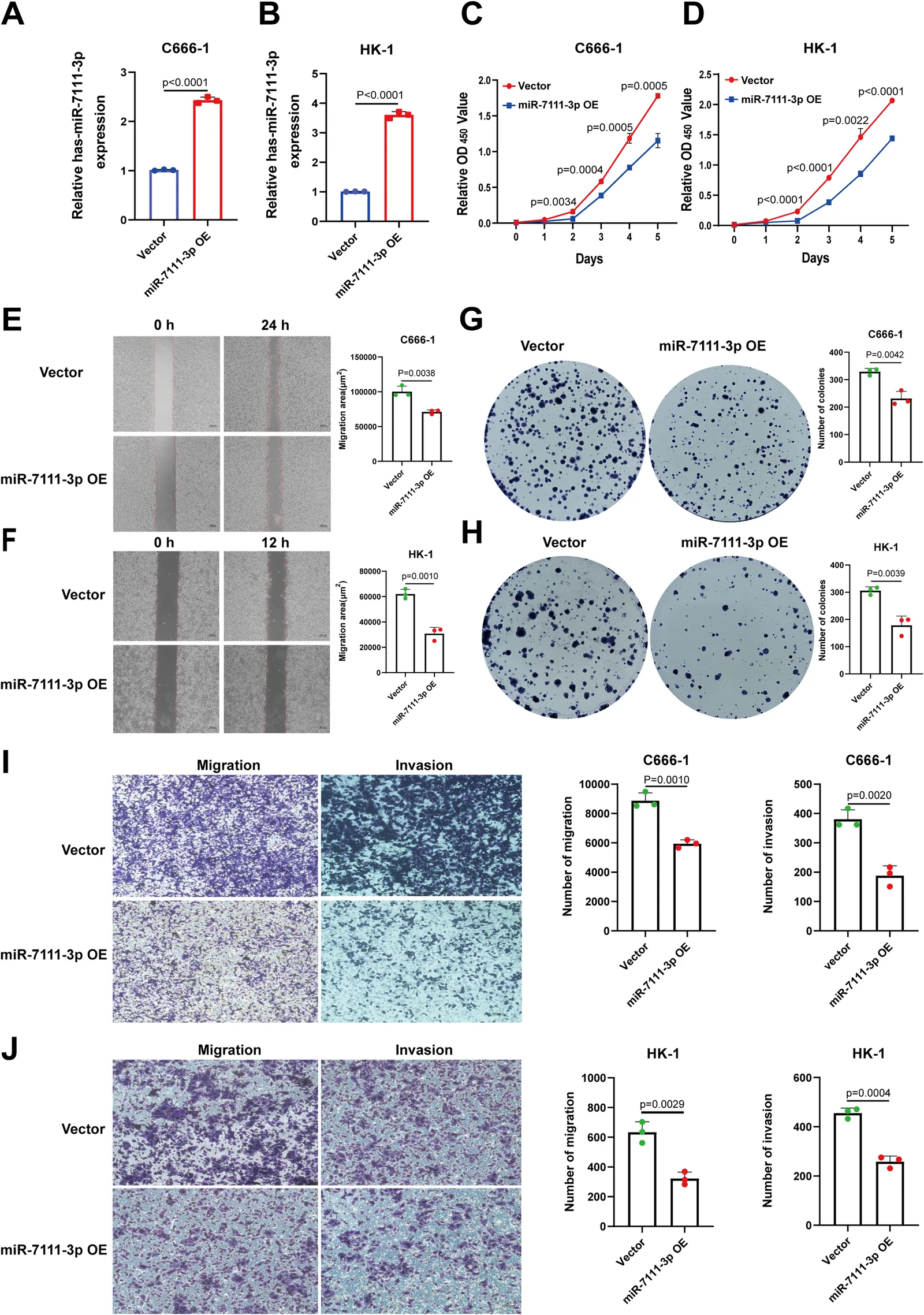
Overexpression of miR-7111-3p inhibits the proliferation, migration, and invasion of NPC cells. (**A**) Transfection efficiency verified by RT-qPCR in C666-1 NPC cells overexpressing miR-7111-3p. (**B**) Transfection efficiency verified by RT-qPCR in HK-1 NPC cells overexpressing miR-7111-3p. (C) Cell proliferation assessed by CCK-8 assay in C666-1 vector/miR-7111-3p OE cells. (**D**) Cell proliferation assessed by CCK-8 assay in HK-1 vector and miR-7111-3p OE cells. (**E**) Cell migration assessed by wound healing assay (50×) and corresponding quantification in C666-1 vector/miR-7111-3p OE cells. (**F**) Cell migration assessed by wound healing assay (50×) and corresponding quantification in HK-1 vector/ miR-7111-3p OE cells. (**G**) Cell proliferation assessed by colony formation assay and corresponding quantification in C666-1 vector/miR-7111-3p OE cells. (**H**) Cell proliferation assessed by colony formation assay and corresponding quantification in HK-1 vector/miR-7111-3p OE cells. (**I**) Cell migration and invasion assessed by Transwell assay (100×) and corresponding quantification in C666-1 vector/ miR-7111-3p OE cells. (**J**) Cell migration and invasion assessed by Transwell assay (100×) and corresponding quantification in HK-1 vector/miR-7111-3p OE cells. All cellular experiments were independently repeated for three biological replicates

Stable miR-7111-3p-overexpressing C666-1 and HK-1 cell lines were established, and overexpression efficiency was confirmed by RT-qPCR (*P* < 0.0001; Fig. 2A, B).

The effect of miR-7111-3p on NPC cell proliferation was subsequently evaluated using CCK-8 and colony formation assays. CCK-8 analysis demonstrated that miR-7111-3p overexpression significantly inhibited the growth of C666-1 cells beginning on day 2 (*P* = 0.0034), with progressively stronger inhibitory effects observed on day 3 (*P* = 0.0004), day 4 (*P* = 0.0005), and day 5 (*P* = 0.0005) (Fig. 2C). Similar results were observed in HK-1 cells, where cell viability was significantly reduced on day 2 (*P* < 0.0001), day 3 (*P* < 0.0001), day 4 (*P* = 0.0022), and day 5 (*P* < 0.0001) following miR-7111-3p overexpression (Fig. 2D). Consistently, colony formation assays showed that ectopic expression of miR-7111-3p significantly reduced clonogenic capacity in both C666-1 and HK-1 cells (*P* = 0.0042 and *P* = 0.0039, respectively; Fig. 2G, H).

To determine whether miR-7111-3p influences NPC cell motility, wound-healing and Transwell assays were performed. Wound-healing assays revealed that miR-7111-3p overexpression significantly impaired migratory ability, as evidenced by reduced wound closure in C666-1 cells (*P* = 0.0038) and HK-1 cells (*P* = 0.0010) compared with vector controls (Fig. 2E, F). Furthermore, Transwell assays demonstrated that miR-7111-3p overexpression markedly suppressed both migration and invasion. In C666-1 cells, the numbers of migrated and invaded cells were significantly decreased (migration: *P* = 0.0010; invasion: *P* = 0.0020; Fig. 2I). Similar inhibitory effects were observed in HK-1 cells (migration: *P* = 0.0029; invasion: *P* = 0.0004; Fig. 2J).

Taken together, these findings demonstrate that miR-7111-3p exerts tumor-suppressive effect in NPC cells by inhibiting cell proliferation, migration and invasion.

### Tumor-promoting effects of miR-7111-3p knockdown

**Fig. 3:**
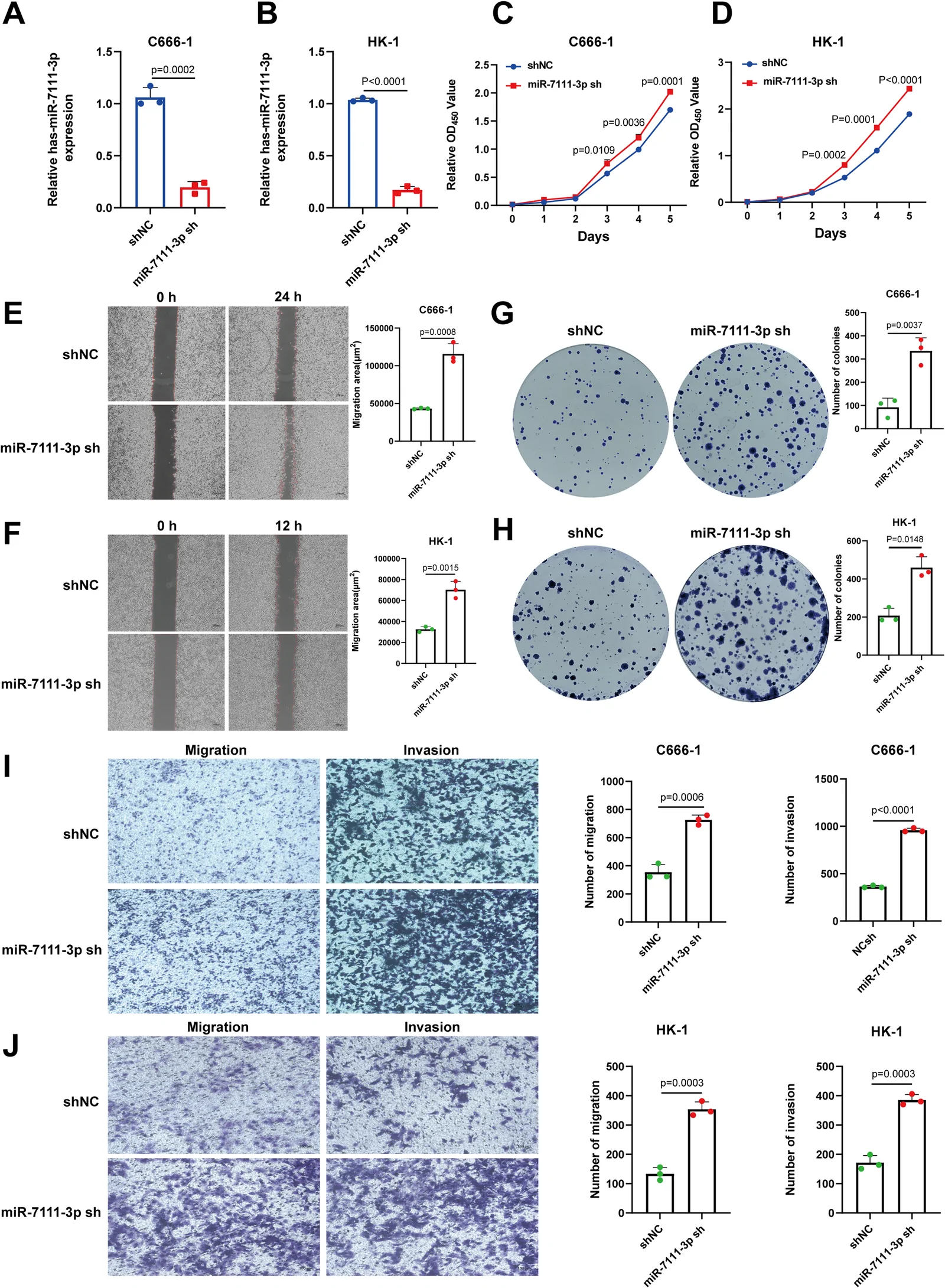
Knockdown of miR-7111-3p promotes the proliferation, migration, and invasion of NPC cells. (**A**) Transfection efficiency verified by RT-qPCR in C666-1 NPC cells with miR-7111-3p knockdown. (**B**) Transfection efficiency verified by RT-qPCR in HK-1 NPC cells with miR-7111-3p knockdown. (**C**) Cell proliferation assessed by CCK-8 assay in C666-1 shNC/miR-7111-3p sh cells. (**D**) Cell proliferation assessed by CCK-8 assay in HK-1 shNC/miR-7111-3p sh cells. (**E**) Cell migration assessed by wound healing assay (50×) and corresponding quantification in C666-1 shNC/miR-7111-3p sh cells. (**F**) Cell migration assessed by wound healing assay (50×) and corresponding quantification in HK-1 shNC/miR-7111-3p sh cells. (**G**) Cell proliferation assessed by colony formation assay and corresponding quantification in C666-1 shNC/miR-7111-3p sh cells. (**H**) Cell proliferation assessed by colony formation assay and corresponding quantification in HK-1 shNC/ miR-7111-3p sh cells. (**I**) Cell migration and invasion assessed by Transwell assay (100×) and corresponding quantification in C666-1 shNC/miR-7111-3p sh cells. (**J**) Cell migration and invasion assessed by Transwell assay (100×) and corresponding quantification in HK-1 shNC/miR-7111-3p sh cells. All cellular experiments were independently repeated for three biological replicates

Loss-of-function experiments confirmed efficient miR-7111-3p silencing in NPC cells (both *P* < 0.05; Fig. 3A, B). Functionally, miR-7111-3p knockdown significantly enhanced cell proliferation, as shown by increased CCK-8 absorbance and colony formation (all *P* < 0.05; Fig. 3C, D, G, H). In addition, wound-healing assays revealed accelerated wound closure after miR-7111-3p knockdown (C666-1, *P* = 0.0008; HK-1, *P* = 0.0015; Fig. 3E, F). Consistently, Transwell assays demonstrated significantly increased migration and invasion capacities in both cell lines (all *P* < 0.05; Fig. 3I, J). These findings further support a tumor-suppressive role of miR-7111-3p in NPC.

### Targeting relationship between miR-7111-3p and APP

**Fig. 4:**
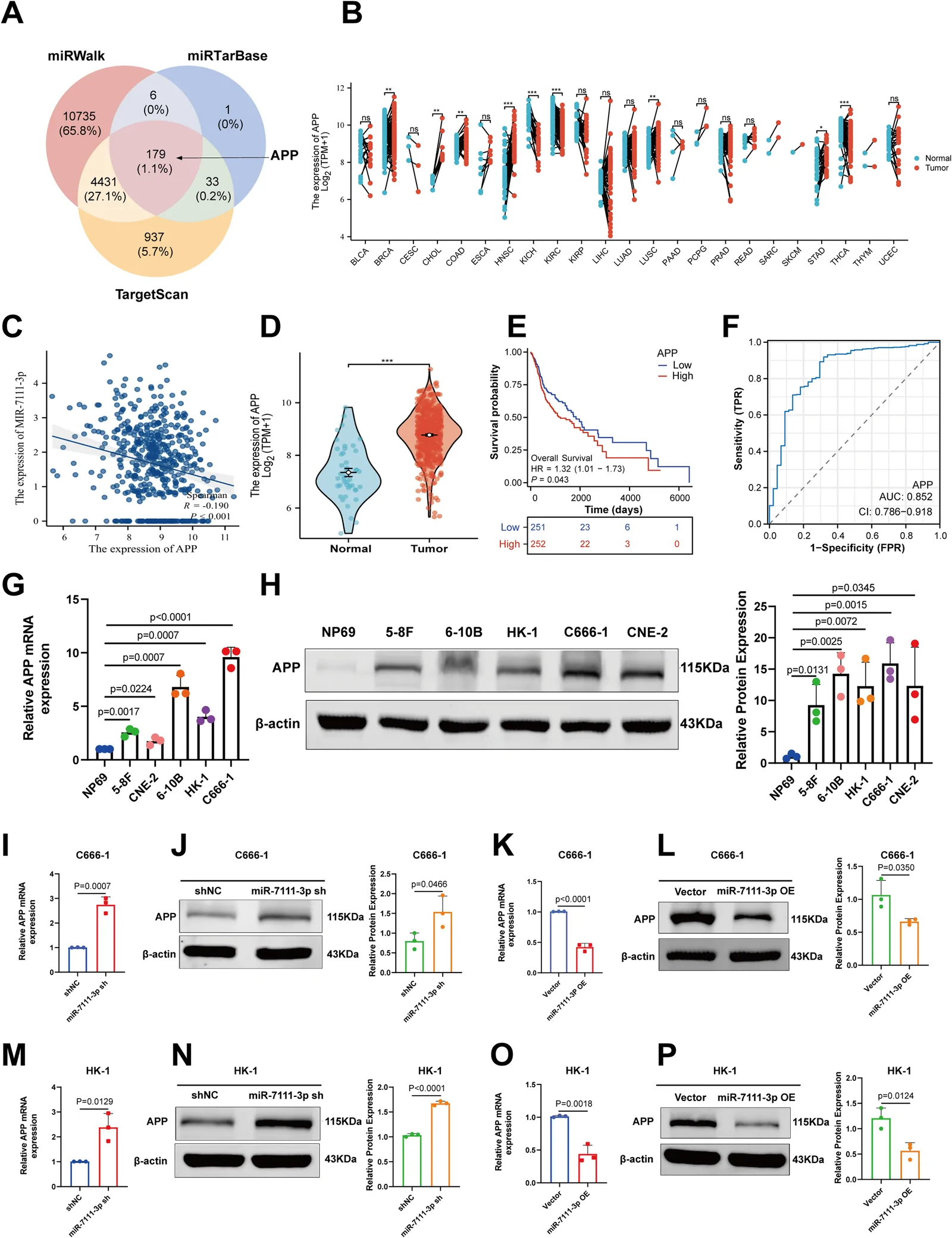
miR-7111-3p suppresses the proliferation, migration, and invasion of NPC cells by targeting APP. (**A**) Venn diagram showing the overlapping predicted target genes of miR-7111-3p from three online databases: miRWalk, miRTarBase, and TargetScan. (**B**) Analysis of target gene APP expression across various cancers using an online database. (**C**) Correlation analysis between miR-7111-3p and APP in head and neck squamous cell carcinoma (HNSCC). (**D**) APP mRNA expression in HNSCC patients. (**E**) Kaplan-Meier survival curve analyzing the prognostic value of APP expression in HNSCC patients. (**F**) Diagnostic ROC curve for APP in HNSCC patients. (**G**) RT-qPCR analysis of APP expression in normal nasopharyngeal epithelial cells and different NPC cell lines. (**H**) Western blot analysis of APP protein expression in normal nasopharyngeal epithelial cells and different NPC cell lines, along with the corresponding statistical chart. (**I, J**) APP expression detected by RT-qPCR and Western blot in C666-1 shNC/miR-7111-3p sh cells. (**K, L**) APP expression detected by RT-qPCR and Western blot in C666-1 vector/miR-7111-3p OE cells. (**M, N**) APP expression detected by RT-qPCR and Western blot in HK-1 shNC/miR-7111-3p sh cells. (**O, P**) APP expression detected by RT-qPCR and Western blot in HK-1 vector/miR-7111-3p OE cells. All cellular experiments were independently repeated for three biological replicates

Bioinformatic analysis identified APP as a candidate target of miR-7111-3p (Fig. 4A). Pan-cancer datasets revealed widespread upregulation of APP in human malignancies (Fig. 4B), and HNSCC data confirmed significantly higher APP expression in tumor tissues compared with normal tissues (*P* < 0.001; Fig. 4D). High APP expression was associated with poor overall survival (HR = 1.32, 95% CI: 1.01–1.73, *P* = 0.043; Fig. 4E) and demonstrated diagnostic value (AUC = 0.852; Fig. 4F).

To determine whether APP is dysregulated in NPC, APP expression was examined in a panel of NPC cell lines. RT-qPCR analysis demonstrated significantly increased APP mRNA expression in 5–8 F (*P* = 0.0017), 6-10B (*P* = 0.0007), HK-1 (*P* = 0.0007), C666-1 (*P* < 0.0001), and CNE-2 cells (*P* = 0.0224) compared with NP69 cells (Fig. 4G). Consistently, Western blot analysis confirmed significantly elevated APP protein levels in NPC cell lines, including 5–8 F (*P* = 0.0131), 6-10B (*P* = 0.0025), HK-1 (*P* = 0.0072), C666-1 (*P* = 0.0015), and CNE-2 cells (*P* = 0.0345) (Fig. 4H).

To further investigate the regulatory relationship between miR-7111-3p and APP, gain- and loss-of-function experiments were performed. Silencing miR-7111-3p significantly increased APP mRNA expression in C666-1 (*P* = 0.0007) and HK-1 cells (*P* = 0.0129) (Fig. 4I, M). Consistent with the transcriptional changes, APP protein expression was markedly elevated following miR-7111-3p knockdown in both C666-1 (*P* = 0.0466) and HK-1 cells (*P* < 0.0001) (Fig. 4J, N). Conversely, overexpression of miR-7111-3p significantly reduced APP expression at both mRNA and protein levels. APP mRNA expression was markedly decreased in C666-1 (*P* < 0.0001) and HK-1 cells (*P* = 0.0018) following miR-7111-3p overexpression (Fig. 4K, O). Similar reductions in protein expression were observed in C666-1 cells (*P* = 0.0350) and HK-1 cells (*P* = 0.0124). (Fig. 4L, P). Dual-luciferase reporter assays confirmed that APP is a direct target of miR-7111-3p (Fig. 6A, B).

**Fig. 5:**
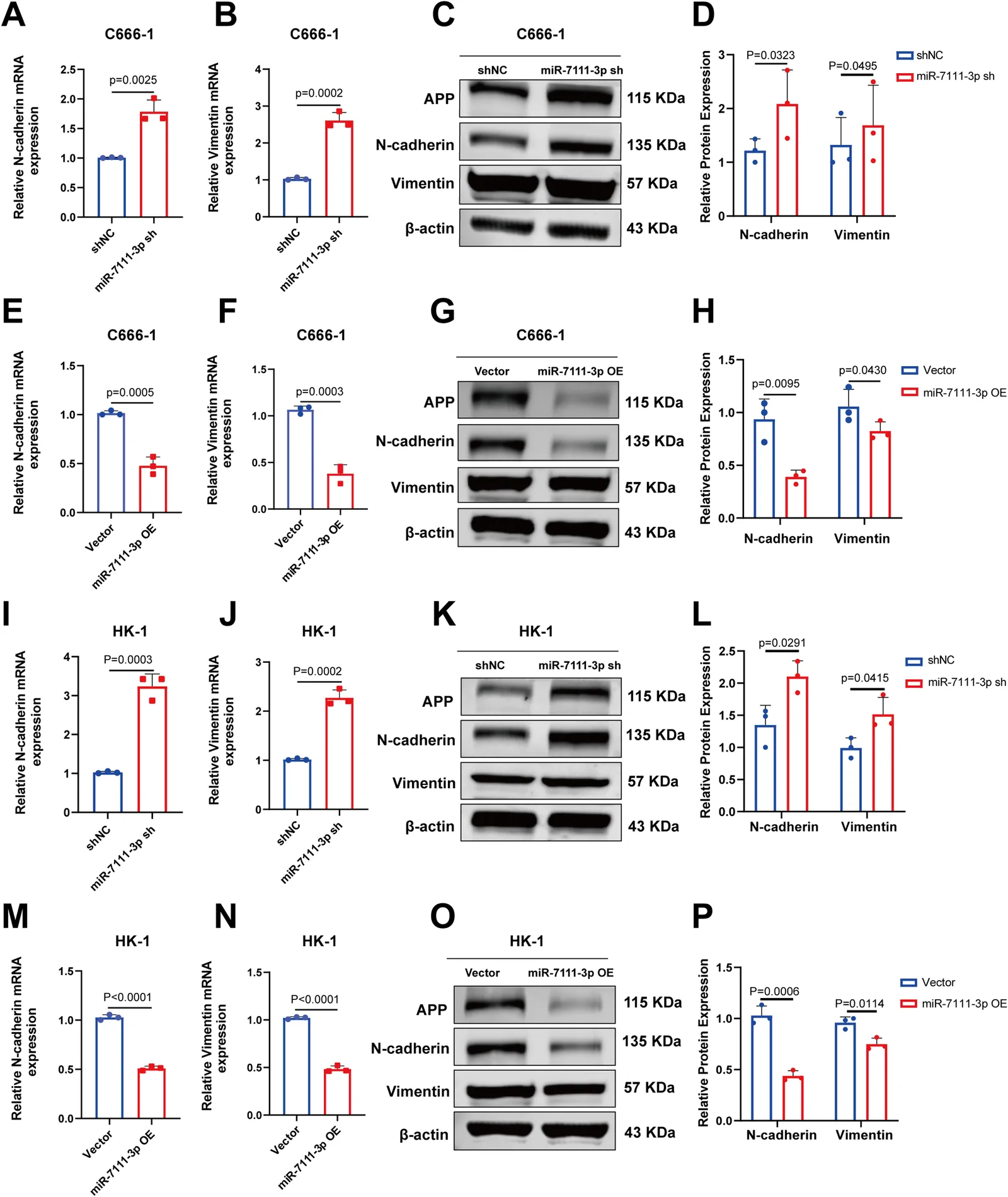
miR-7111-3p induces Epithelial-mesenchymal transition (EMT) in NPC cells by negatively regulating APP expression. (**A, B**) mRNA expression levels of EMT markers (N-cadherin, Vimentin) detected by RT-qPCR in C666-1 shNC/miR-7111-3p sh cells. (**C, D**) Western blot analyses of APP and EMT marker (N-cadherin, Vimentin) protein expression in C666-1 shNC and miR-7111-3p sh cells, with the accompanying statistical chart. (**E, F**) mRNA expression levels of EMT markers (N-cadherin, Vimentin) detected by RT-qPCR in C666-1 vector/miR-7111-3p OE cells. (**G, H**) Western blot analyses of APP and EMT marker (N-cadherin, Vimentin) protein expression in C666-1 vector and miR-7111-3p OE cells, with the accompanying statistical chart. (**I, J**) mRNA expression levels of EMT markers (N-cadherin, Vimentin) detected by RT-qPCR in HK-1 shNC and miR-7111-3p sh cells. (**K, L**) Western blot analyses of APP and EMT marker (N-cadherin, Vimentin) protein expression in HK-1 shNC and miR-7111-3p sh cells, with the accompanying statistical chart. (**M, N**) mRNA expression levels of EMT markers (N-cadherin, Vimentin) detected by RT-qPCR in HK-1 vector/miR-7111-3p OE cells. (**O, P**) Western blot analyses of APP and EMT marker (N-cadherin, Vimentin) protein expression in HK-1 vector/miR-7111-3p OE cells, with the accompanying statistical chart. All cellular experiments were independently repeated for three biological replicates

### miR-7111-3p regulates EMT in NPC cells via targeting APP

To characterize the regulatory role of miR-7111-3p in EMT of NPC, we assessed the expression of EMT-associated markers following miR-7111-3p knockdown and overexpression in C666-1 and HK-1 cells. In C666-1 cells, miR-7111-3p knockdown robustly elevated the transcript abundance of mesenchymal markers N-cadherin (*P* = 0.0025; Fig. 5A) and Vimentin (*P* = 0.0002; Fig. 5B) compared with shNC controls. Consistently, Western blot analysis confirmed that protein expression of N-cadherin and Vimentin was significantly upregulated following miR-7111-3p knockdown (N-cadherin, *P* = 0.0323; Vimentin, *P* = 0.0495; Fig. 5C, D). Conversely, miR-7111-3p overexpression significantly reduced N-cadherin (*P* = 0.0005; Fig. 5E) and Vimentin (*P* = 0.0003; Fig. 5F) mRNA expression. These findings were further validated at the protein level, where both N-cadherin and Vimentin were significantly downregulated in miR-7111-3p-overexpressing cells (N-cadherin, *P* = 0.0095; Vimentin, *P* = 0.0430; Fig. 5G, H).

**Fig. 6:**
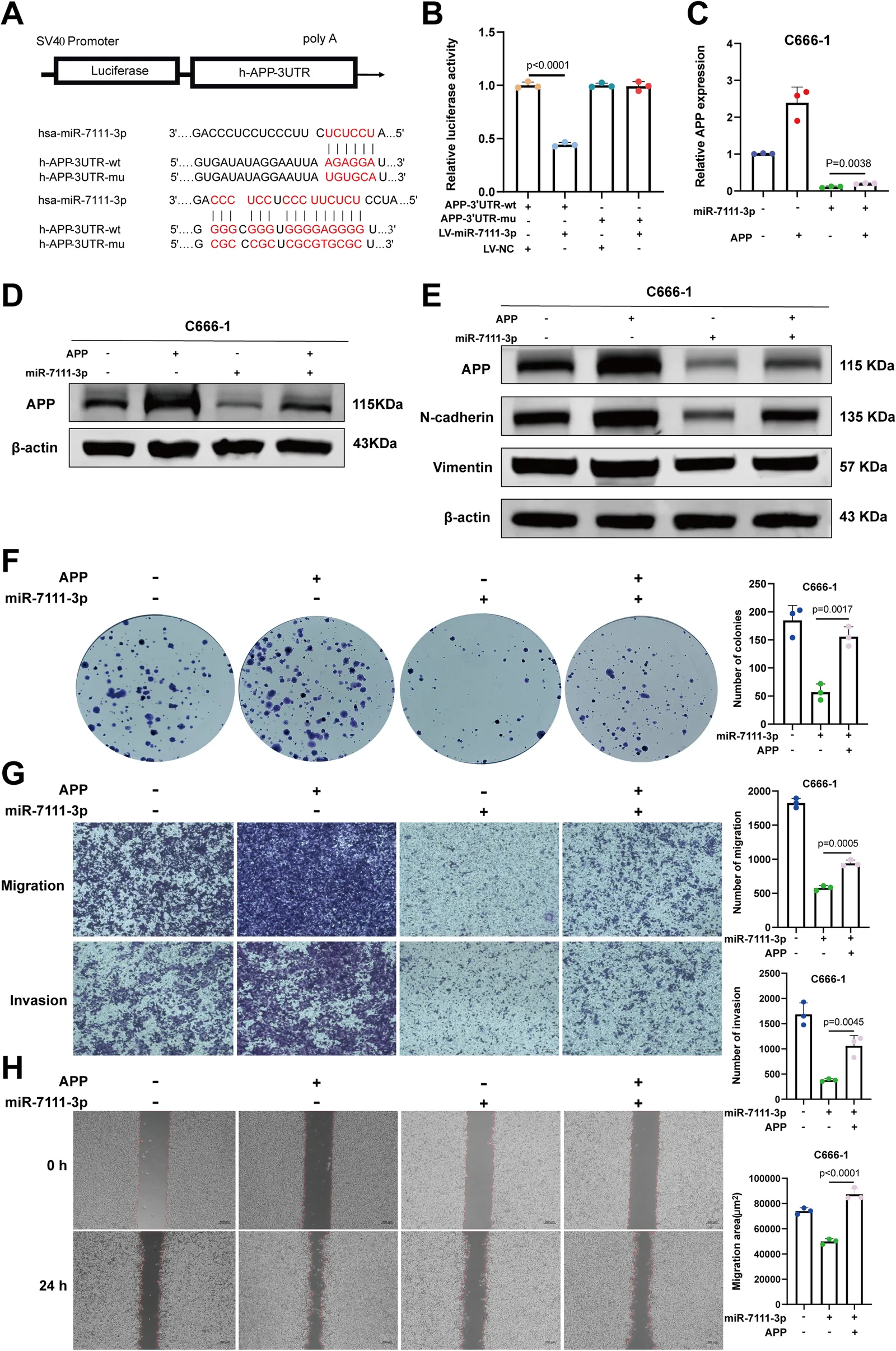
Overexpression of APP reverses the suppressive effects of miR-7111-3p on proliferation, migration, and invasion in C666-1 NPC cells. (**A**) Schematic diagram of the predicted binding site of hsa-miR-7111-3p on the h-APP-3’UTR. (**B**) Dual-luciferase reporter assay verifying the interaction between hsa-miR-7111-3p and the h-APP-3’UTR. (**C, D**) APP expression detected by RT-qPCR and Western blot in C666-1 cells under four conditions: vector + APP NC, vector + APP OE, miR-7111-3p OE + APP NC, and miR-7111-3p OE + APP OE. (**E**) Protein expression levels of APP and EMT markers (N-cadherin, Vimentin) detected by Western blot. (**F**) Colony formation assay. (**G**) Representative images of Transwell migration and invasion assays (100×). (**H**) Representative images of wound healing assay (50×). All cellular experiments were independently repeated for three biological replicates

Identical expression patterns were reproduced in HK-1 cells. Knockdown of miR-7111-3p significantly increased N-cadherin (*P* = 0.0003; Fig. 5I) and Vimentin (*P* = 0.0002; Fig. 5J) mRNA expression compared with shNC controls. Correspondingly, protein expression levels were also significantly elevated following miR-7111-3p silencing (N-cadherin, *P* = 0.0291; Vimentin, *P* = 0.0415; Fig. 5K, L). In contrast, miR-7111-3p overexpression markedly suppressed EMT marker expression at both mRNA and protein levels in HK-1 cells, with significant reductions observed for N-cadherin (*P* < 0.0001; Fig. 5M) and Vimentin (*P* < 0.0001; Fig. 5N) mRNA expression, as well as protein levels (N-cadherin, *P* = 0.0006; Vimentin, *P* = 0.0114; Fig. 5O, P). To complete EMT marker analysis in vitro, we detected E-cadherin at both the transcriptional and protein levels in C666-1 and HK-1 cell lines (Supplementary Figure S1). The results showed that miR-7111-3p knockdown significantly reduced E-cadherin mRNA and protein expression, whereas miR-7111-3p overexpression markedly increased E-cadherin expression in both cell lines, with all P values < 0.05.

In addition, APP protein expression showed a consistent trend with EMT marker changes across all experimental conditions, further supporting its involvement in miR-7111-3p-mediated regulatory effects on EMT. Collectively, these results demonstrate that miR-7111-3p inhibits EMT progression in NPC cells, at least in part through direct regulation of APP.

### APP overexpression reverses the tumor-suppressive effects of miR-7111-3p

To further determine whether APP is a functional downstream effector of miR-7111-3p, rescue experiments were performed in C666-1 and HK-1 cells. To evaluate whether APP could reverse the biological effects induced by miR-7111-3p, APP was ectopically overexpressed in miR-7111-3p-overexpressing NPC cells. Western blot analysis confirmed successful modulation of APP expression across experimental groups (Fig. 6C, D).

Functional assays showed that APP overexpression significantly restored colony formation ability suppressed by miR-7111-3p in C666-1 cells (*P* = 0.0017; Fig. 6F). Similarly, APP overexpression markedly reversed the inhibitory effects of miR-7111-3p on cell migration (*P* = 0.0005) and invasion (*P* = 0.0045) (Fig. 6G). Wound-healing assays further confirmed that APP restoration significantly increased migratory capacity in miR-7111-3p-overexpressing cells (*P* < 0.0001; Fig. 6H). Importantly, APP overexpression significantly reversed the suppressive effects of miR-7111-3p on EMT-associated proteins. Specifically, N-cadherin and Vimentin levels were markedly increased (Fig. 6E), while E-cadherin expression was decreased following APP restoration (Fig. S2A), indicating a re-activation of EMT.

**Fig. 7:**
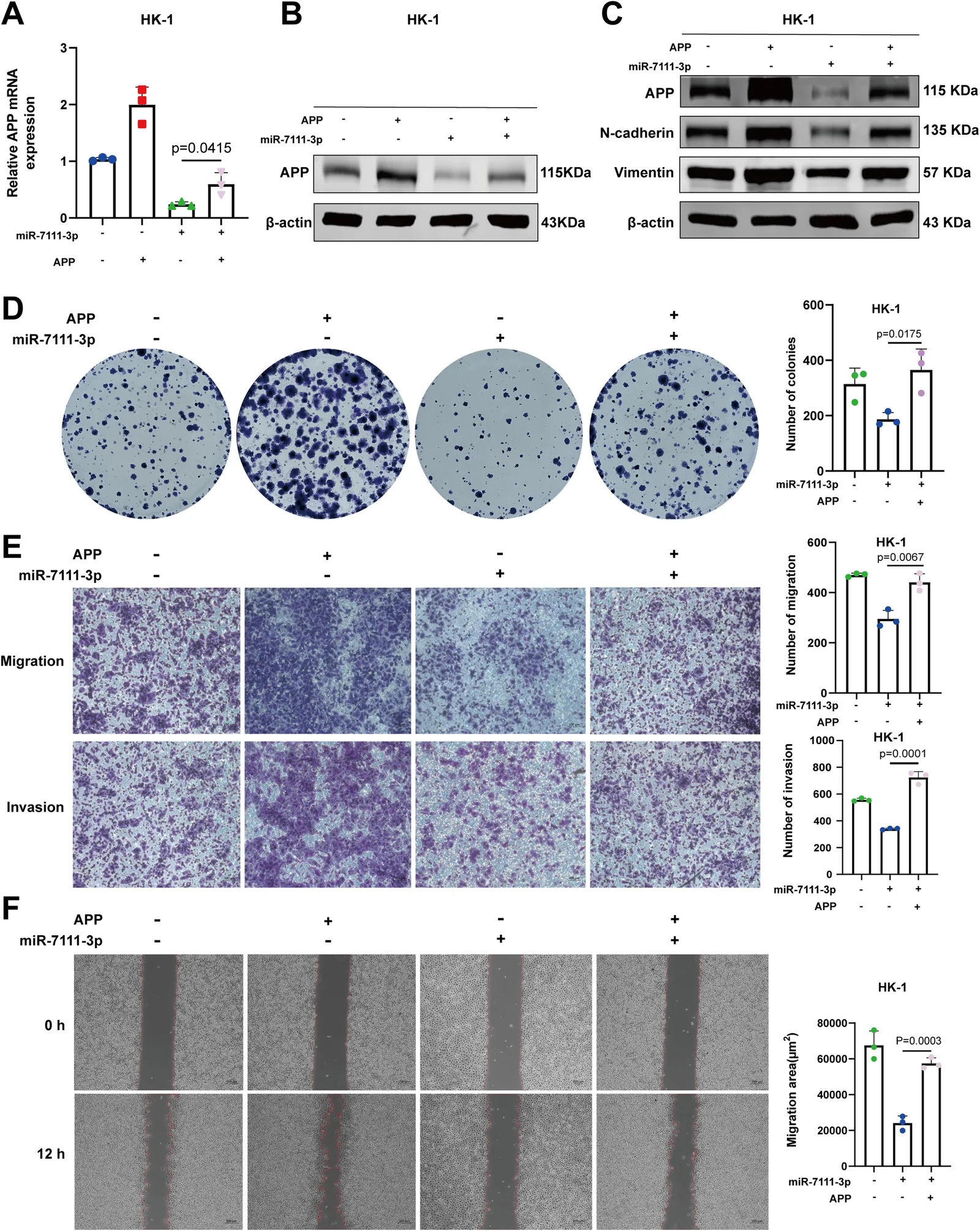
Overexpression of APP reverses the suppressive effects of miR-7111-3p on proliferation, migration, and invasion in HK-1 NPC cells. (**A, B**) APP expression detected by RT-qPCR and Western blot in HK-1 cells under four conditions: vector + APP NC, vector + APP OE, miR-7111-3p OE + APP NC, and miR-7111-3p OE + APP OE. (**C**) Protein expression levels of APP and EMT markers (N-cadherin, Vimentin) detected by Western blot. (**D**) Colony formation assay and corresponding quantification. (**E**) Representative images and quantification of Transwell migration and invasion assays (100×). (**F**) Representative images and quantification of wound healing assay (50×)

Consistently, in HK-1 cells, APP overexpression significantly reversed the suppressive effects of miR-7111-3p on APP protein expression (Fig. 7A, B). Functionally, APP restoration significantly promoted colony formation (*P* = 0.0175; Fig. 7D), migration (*P* = 0.0067; Fig. 7E), and invasion (*P* = 0.0001; Fig. 7E) compared with miR-7111-3p overexpression alone. Wound-healing assays further confirmed significant recovery of migratory capacity following APP overexpression (*P* = 0.0003; Fig. 7F). Importantly, APP restoration significantly reversed the inhibitory effects of miR-7111-3p on EMT-related proteins. Western blot analysis demonstrated that E-cadherin expression was significantly decreased (Fig. S2B), whereas N-cadherin and Vimentin were significantly increased following APP overexpression (Fig. 7C), indicating EMT reactivation.

Collectively, these findings demonstrate that APP is a critical functional mediator of miR-7111-3p in regulating NPC cell proliferation, migration, and invasion.

### Isolation, characterization, and uptake of EVs

**Fig. 8:**
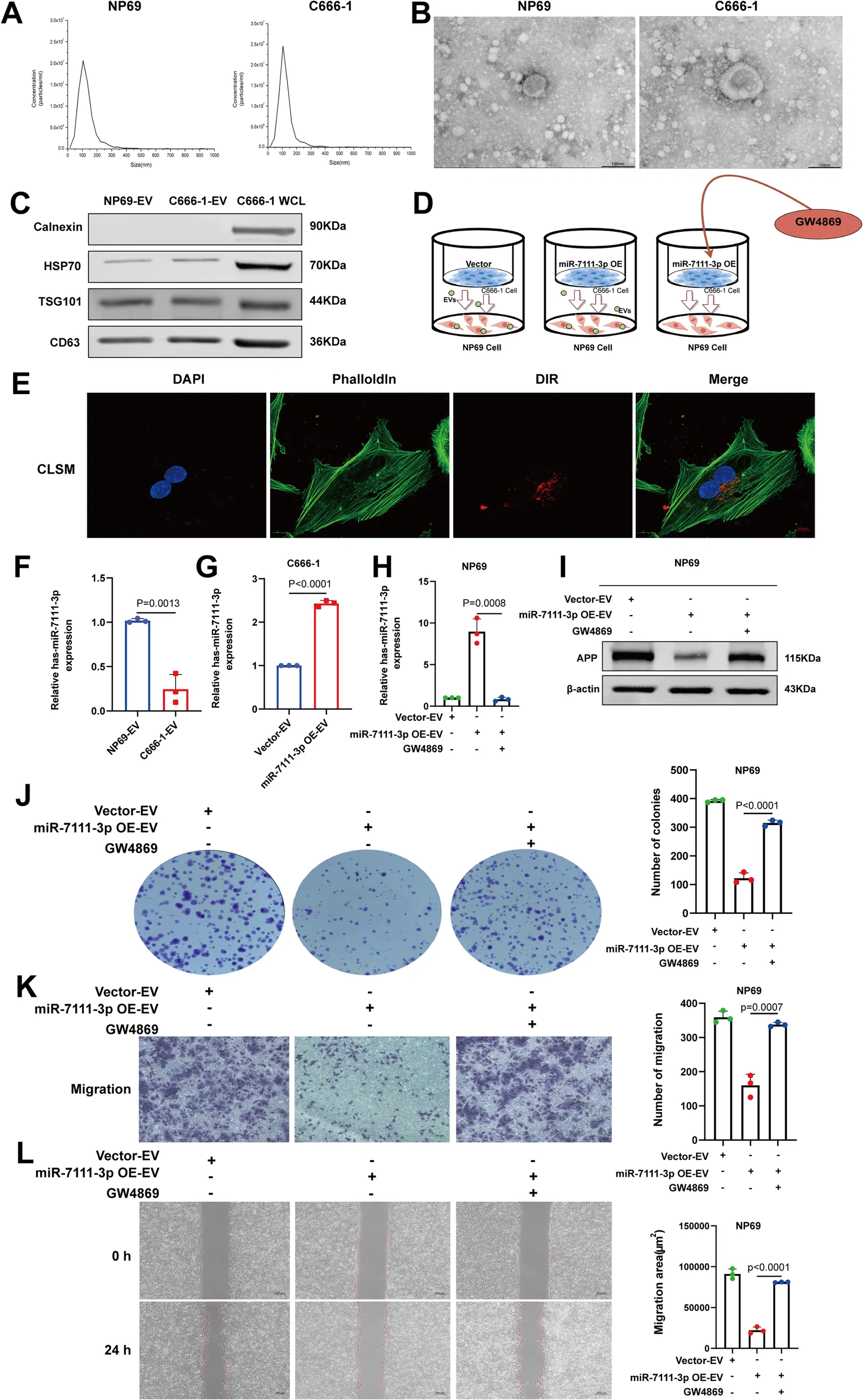
NPC-derived extracellular vesicles (EVs) with low miR-7111-3p enhance NP69 cell proliferation and migration by upregulating APP. (**A**) Nanoparticle tracking analysis (NTA) of EVs size distribution. (**B**) Morphology of EVs observed by transmission electron microscopy (TEM). (**C**) Western blot analysis of EVs marker proteins. (**D**) Schematic diagram of the co-culture mechanism. (**E**) Uptake of DIR-labeled EVs (red) by normal nasopharyngeal epithelial NP69 cells. (**F**) RT-qPCR analysis of miR-7111-3p expression in EVs derived from NP69 and C666-1 cells. (**G**) RT-qPCR analysis of miR-7111-3p expression in EVs derived from C666-1 vector and miR-7111-3p OE cells. (**H**) RT-qPCR analysis of miR-7111-3p expression in NP69 cells after co-culture with C666-1 vector, miR-7111-3p OE, or miR-7111-3p OE plus the EV inhibitor GW4869. (**I**) Western blot analysis of APP expression in NP69 cells after co-culture under the conditions described in (**H**). (**J**) Colony formation assay and corresponding quantification assessing the proliferation of NP69 cells after co-culture. (**K**) Transwell assay and corresponding quantification assessing the migration of NP69 cells after co-culture. (**L**) Wound healing assay and corresponding quantification assessing the migration of NP69 cells after co-culture. All cellular experiments were independently repeated for three biological replicates

EVs were isolated from NP69 and C666-1 cells using differential ultracentrifugation. TEM revealed typical cup-shaped vesicle morphology (Fig. 8B). NTA showed that EVs exhibited a size distribution predominantly ranging from approximately 100–200 nm (Fig. 8A). Western blot analysis confirmed enrichment of EV markers CD63, TSG101, and HSP70, while the negative marker Calnexin was not detected, indicating successful isolation of purified EVs (Fig. 8C).

### Downregulated miR-7111-3p in EVs attenuates inhibition of APP, promoting NP69 cells proliferation and migration

A co-culture system was established as shown in Fig. 8D, where NP69 cells were co-cultured for 48 h with C666-1 Vector, miR-7111-3p OE, or OE + GW4869 inhibitor group cells. Fluorescence imaging confirmed EV uptake by NP69 cells (Fig. 8E).

miR-7111-3p levels were significantly lower in EVs derived from C666-1 cells compared with NP69-derived EVs (*P* = 0.0013; Fig. 8F), whereas miR-7111-3p overexpression increased its EV abundance (*P* < 0.0001; Fig. 8G). Following EV uptake, NP69 recipient cells treated with EVs from miR-7111-3p-overexpressing C666-1 cells exhibited significantly increased intracellular miR-7111-3p levels compared with vector-EV controls (*P* = 0.0008; Fig. 8H). This effect was markedly abolished by the EV secretion inhibitor GW4869, confirming EV-dependent transfer.

Functionally, EVs enriched with miR-7111-3p significantly suppressed phenotypes in NP69 cells. Colony formation ability was significantly reduced (*P* < 0.0001; Fig. 8J), while migration ability was also markedly decreased (*P* = 0.0007; Fig. 8K). Wound-healing assays further confirmed impaired migratory capacity, which was partially reversed by GW4869 treatment (*P* < 0.0001; Fig. 8L). Mechanistically, Western blot analysis revealed that APP expression was markedly suppressed in NP69 cells incubated with miR-7111-3p-enriched EVs, whereas GW4869 restored APP expression levels (Fig. 8I). Inhibition of EV secretion via GW4869 reversed the phenotypic changes of NP69 cells induced by co-culture with NPC cells, preliminarily suggesting NPC-derived EVs mediate intercellular communication between NPC and normal nasopharyngeal epithelial cells. To eliminate interference from GW4869 off-target effects and verify two core questions: whether miR-7111-3p is encapsulated within EVs, and whether purified EVs carrying miR-7111-3p directly alter recipient cell phenotypes. These corroborative datasets are fully presented in Supplementary Figures S3 and S4.

First, an RNase protection assay was conducted to distinguish intravesicular miR-7111-3p from free extracellular miRNA (Fig. S3A-B). Isolated EVs were divided into three groups: PBS control, RNase A single treatment, and combined Triton X-100 + RNase A treatment. RT-qPCR showed that miR-7111-3p expression remained unchanged between the PBS control and RNase A-only groups (ns). In contrast, miR-7111-3p abundance was nearly eliminated after EV membrane permeabilization with Triton X-100 followed by RNase digestion (*p* < 0.0001). This confirms miR-7111-3p is packaged inside EVs, protected by the lipid bilayer from extracellular RNase degradation. Then, we performed quantitative purified EV add-back experiments (Fig. S3C). NP69 cells were incubated with equal particle amounts of PBS, EVs secreted by Vector-transfected C666-1 cells (EVs-Vector), or EVs secreted by miR-7111-3p-overexpressing C666-1 cells (EVs-miR-7111-3p OE). As illustrated in Supplementary Figure S4, compared with PBS and EVs-Vector groups, NP69 cells treated with EVs-miR-7111-3p OE exhibited significantly elevated intracellular miR-7111-3p levels (Fig. S4A), downregulated APP mRNA and protein (Fig. S4B, C), alongside suppressed colony formation and migration capacity (Fig. S4D, E, F). These results directly prove that EV-delivered miR-7111-3p is the functional cargo driving APP suppression and proliferation and migration in recipient NP69 cells.

These results indicate that NPC-derived EVs carrying low levels of miR-7111-3p can promote the proliferation and migration of normal nasopharyngeal epithelial NP69 cells by attenuating the inhibition of APP, leading to APP upregulation and enhanced proliferative and migratory potential of NP69 cells. However, enhanced cell motility and proliferation alone are insufficient to confirm the malignant transformation of normal nasopharyngeal epithelial cells.

### miR-7111-3p inhibits the growth of NPC subcutaneous xenograft tumors by downregulating APP expression

**Fig. 9:**
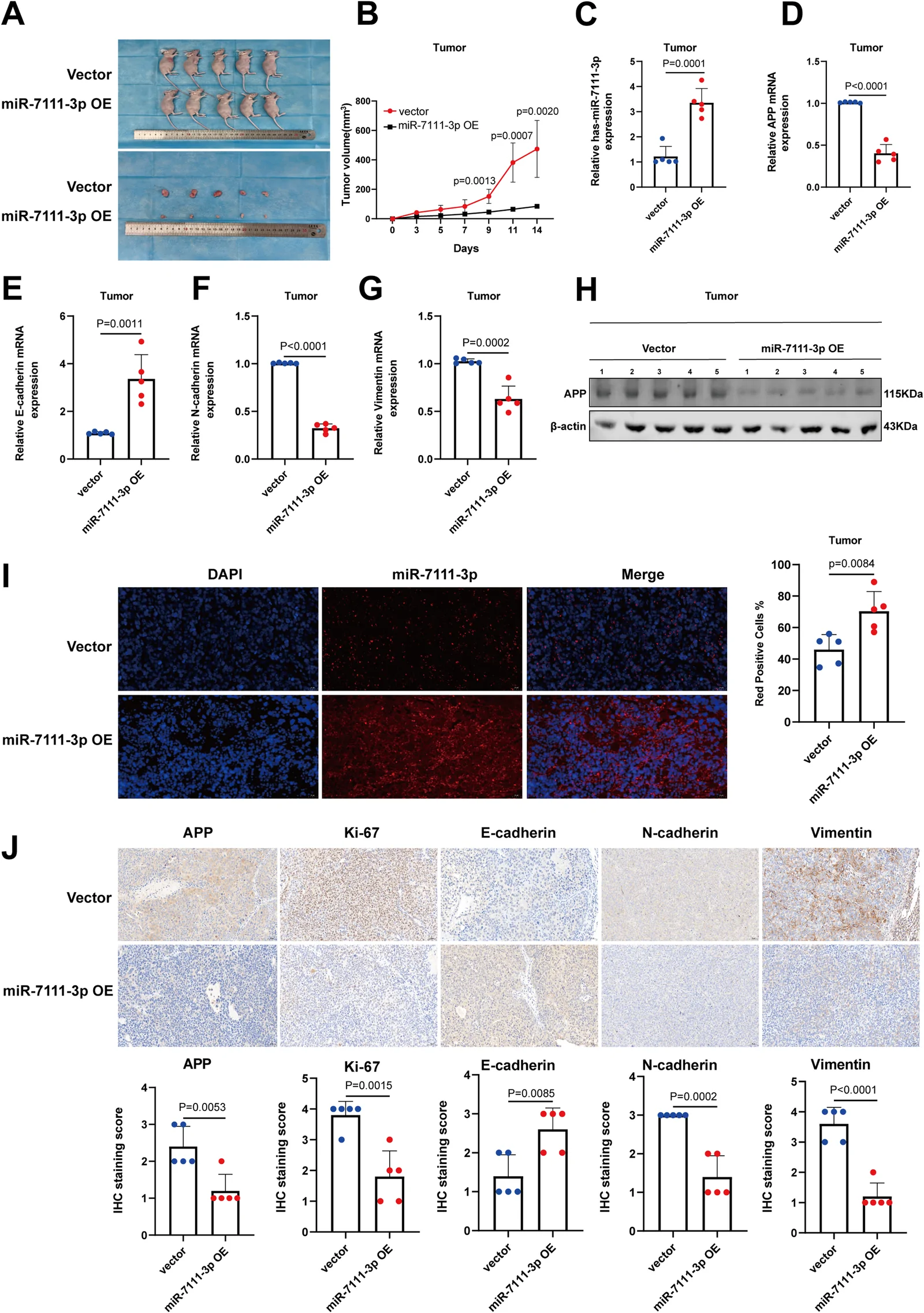
miR-7111-3p inhibits NPC tumor growth in vivo by negatively regulating APP expression. (**A**) Gross view of xenograft tumors in nude mice; (**B**) Statistical chart of tumor volumes in the vector group and miR-7111-3p OE group. (**C**) RT-qPCR detection of miR-7111-3p expression in tumor tissues from the vector group and miR-7111-3p OE group. (**D**) RT-qPCR detection of APP mRNA expression in tumor tissues from the vector group and miR-7111-3p OE group. (**E-G**) RT-qPCR detection of mRNA expression of E-cadherin, N-cadherin, and Vimentin in tumor tissues from the vector group and miR-7111-3p OE group. (**H**) Western blot detection of APP protein expression in tumor tissues from the vector group and miR-7111-3p OE group. (**I**) Fluorescence in situ hybridization (FISH) detecting miR-7111-3p expression in tumor tissues from the vector group and miR-7111-3p OE group (400×). (J)Statistical chart of FISH testing. (**K**) Immunohistochemistry (IHC) detection of protein expression of APP, Ki-67, E-cadherin, N-cadherin, and Vimentin (200×). All cellular experiments were independently repeated for three biological replicates.; in vivo xenograft experiment included five nude mice per experimental group

In xenograft models, miR-7111-3p overexpression significantly reduced tumor growth (*P* < 0.05; Fig. 9A, B). Tumor tissues showed increased miR-7111-3p (*P* = 0.0001; Fig. 9C) and decreased APP expression (*P* < 0.0001; Fig. 9D), confirming successful modulation of the axis in vivo. EMT analysis showed increased E-cadherin (*P* = 0.0011; Fig. 9E) and decreased N-cadherin (*P* < 0.0001; Fig. 9F) and Vimentin in the miR-7111-3p group (*P* = 0.0002; Fig. 9G). Western blot analysis further confirmed that APP protein expression was significantly reduced in tumor tissues from the miR-7111-3p overexpression group compared with controls (Fig. 9H).

FISH results showed higher miR-7111-3p fluorescence signal intensity in the miR-7111-3p OE group compared to the vector group (Fig. 9I). In addition, IHC analysis demonstrated that Ki-67, N-cadherin, and Vimentin expression levels were significantly decreased, whereas E-cadherin expression was significantly increased in the miR-7111-3p overexpression group compared with the control group (Ki-67, *P* = 0.0015; N-cadherin, *P* = 0.0002; Vimentin, *P* < 0.0001; E-cadherin, *P* = 0.0085; Fig. 9J). These findings are consistent with in vitro results, demonstrating that miR-7111-3p suppresses NPC tumor growth via APP-mediated EMT regulation. Notably, the subcutaneous model cannot reflect distant metastasis of NPC.

## Discussion

This study systematically investigated the expression dynamics and biological functions of miR-7111-3p during NPC progression. To our knowledge, revealed its bidirectional regulatory role in NPC proliferation, migration, invasion, and EMT through an EV-mediated APP targeting axis. By integrating in vitro functional assays, in vivo xenograft models, and clinical sample analyses, we propose that the miR-7111-3p/APP axis represents a critical regulatory link in NPC malignant progression, in which EVs serve as key mediators of intercellular signal transmission.

In recent years, a growing body of evidence has highlighted the critical role of miRNAs in the initiation, progression, and therapeutic response of NPC. For example, transactivated MIR106A-5p has been reported to inhibit autophagy by targeting BTG3, thereby activating MAPK signaling and promoting malignant phenotypes in NPC [18]. In addition, accumulating evidence suggests that radioresistance in NPC is closely associated with enhanced invasive and metastatic potential [19]. Clinically, one of the major causes of recurrence and metastasis after radiotherapy is the incomplete eradication of highly radioresistant tumor cell subclones within the primary lesion, which subsequently survive, proliferate, accumulate genetic alterations, and eventually evolve into local recurrence or distant metastasis [20]. Moreover, miR-197-3p has been shown to be closely associated with radioresistance, apoptosis, proliferation, migration, and overall survival in NPC [21]. Collectively, these findings indicate a close and intrinsic relationship between radioresistance and tumor invasion/metastasis. Based on GEO dataset analysis and clinical sample detection, our study found that miR-7111-3p is significantly downregulated in NPC serum samples and cell lines, with even lower expression observed in radioresistant patients. This suggests that miR-7111-3p is downregulated in radioresistant NPC samples compared with radiosensitive cases, indicating a potential association between miR-7111-3p expression and NPC radioresistance. However, whether miR-7111-3p directly regulates radiosensitivity requires further investigation.

EMT is associated with malignant phenotypes in various cancers, including enhanced proliferation, invasion, and metastasis. Increasing evidence has demonstrated that multiple cellular miRNAs play essential roles in regulating EMT-related processes [22, 23]. For instance, miR-28-3p has been reported to promote EMT by downregulating Nm23-H1, thereby enhancing NPC cell migration and invasion [24]. Conversely, Huang et al. demonstrated that miR-34a suppresses TGF-β-induced EMT, invasion, and migration in NPC cells by directly targeting SMAD4 [25]. These studies highlight the therapeutic potential of miRNAs in NPC. In the present study, we found that miR-7111-3p overexpression significantly inhibited NPC cell proliferation, migration, invasion, and EMT, as evidenced by decreased expression of N-cadherin and Vimentin and increased expression of E-cadherin, indicating EMT suppression. In contrast, miR-7111-3p knockdown produced the opposite effects. In vivo animal experiments further confirmed the tumor-suppressive role of miR-7111-3p. Collectively, these bidirectional functional results provide strong evidence supporting the tumor-suppressive role of miR-7111-3p in NPC.

APP is a transmembrane glycoprotein that has been extensively studied in neurodegenerative disorders such as Alzheimer’s disease. However, accumulating evidence indicates that APP also exerts oncogenic functions in multiple cancer types [26, 27]. Previous studies have reported that APP is overexpressed in breast cancer, prostate cancer, and head and neck squamous cell carcinoma, where it is associated with enhanced cell migration, invasion, and poor clinical outcomes [28–30]. In the present study, we identified APP as a direct target of miR-7111-3p through integrated bioinformatics prediction, clinical validation, and functional assays. We further confirmed that APP is significantly upregulated in NPC cells, consistent with an EMT-activated phenotype. Importantly, APP overexpression partially rescued the inhibitory effects of miR-7111-3p on NPC cell proliferation, migration, and invasion, indicating that APP acts as a key downstream effector in this regulatory axis. Mechanistically, previous studies have suggested that APP could promote EMT through activation of the MAPK/ERK signaling pathway, which subsequently downregulates tissue inhibitor of metalloproteinase 2 (TIMP2) and facilitates the acquisition of mesenchymal traits in epithelial cells [11]. However, we did not directly investigate MAPK/ERK signaling in our experimental models. The linkage between APP, MAPK/ERK signaling and EMT described above is only a hypothesis inferred from published literature, rather than a mechanism confirmed by the present study. Further investigations are required to elucidate the precise downstream signaling mechanisms by which APP regulates EMT in NPC.

EVs are key mediators of intercellular communication and are capable of transferring bioactive molecules, including miRNAs, lncRNAs, circRNAs, and proteins, to recipient cells. Through this process, EVs contribute to tumor microenvironment remodeling, immune evasion, drug resistance, and metastasis [31–33]. Previous studies have highlighted the dual roles of EVs in NPC. For example, Wan et al. reported that exosomes derived from miR-34c-transfected mesenchymal stem cells suppress NPC invasion, migration, proliferation, and EMT [19]. Conversely, exosomal miR-18a-5p derived from NPC cells has been shown to promote proliferation, migration, invasion, and EMT in NPC [34]. In addition, NPC-derived exosomes have been reported to facilitate tumor progression via the miR-99a-5p/BAZ2A axis [35], further supporting the functional importance of EV-mediated signaling in NPC. Our research found that miR-7111-3p levels were significantly reduced in EVs derived from NPC cells compared with those from normal nasopharyngeal epithelial cells. This reduction weakens its inhibitory effect on APP in recipient cells, leading to APP upregulation. Through EV characterization, GW4869 inhibition assays, RNase protection experiments, and EV add-back functional studies, our integrated data demonstrate that C666-1-derived EVs deliver functional miR-7111-3p to NP69 recipient cells, thereby suppressing APP expression and inhibiting proliferative and migratory capacities. This result suggests that EVs not only transmit pro-tumor signals between tumor cells but may also deliver tumor-suppressive signals. However, in NPC, the reduction of miR-7111-3p within EVs implies a loss of this tumor-suppressive signal, conversely promoting tumor progression. Tumor-cell-derived EV carrying low miR-7111-3p only increases the proliferative and migratory capacity of normal NP69 cells through derepression of APP.

Serum miRNAs have attracted considerable attention as liquid biopsy biomarkers due to their high stability and non-invasive accessibility [36, 37]. In this study, our preliminary analysis in a small cohort revealed decreased circulating miR-7111-3p levels in NPC patients compared with healthy controls, providing only an initial indication for further biomarker investigation. Importantly, the current sample size is insufficient to perform reliable ROC curve analysis or to draw definitive conclusions regarding the diagnostic or prognostic performance of miR-7111-3p and APP. Therefore, large-scale prospective clinical studies are required to systematically evaluate whether combined detection of circulating miR-7111-3p and APP could serve as a clinically valuable diagnostic or prognostic biomarker, potentially using ddPCR or high-throughput sequencing in future investigations.

Restoration of miR-7111-3p function or inhibition of APP signaling may represent a promising therapeutic strategy for NPC. Currently, strategies such as miRNA mimics, miRNA expression vectors, and small interfering RNAs (siRNAs) targeting genes have entered preclinical development stage in various cancers [38, 39]. Among these, EV-based miRNA delivery systems offer distinct advantages, including high biocompatibility and the ability to cross biological barriers. In this context, our findings provide theoretical support for the development of EV-based delivery systems carrying miR-7111-3p for targeted therapy of NPC. Furthermore, to our knowledge, no previous studies have reported a direct link between EVs and the miR-7111-3p/APP regulatory axis in NPC, highlighting the mechanistic novelty of the present study.

Although this study provides systematic insights into the molecular mechanisms and biological functions of miR-7111-3p in NPC, several limitations should be acknowledged. First, the number of clinical serum specimens (11 NPC patients and 11 healthy subjects) is relatively limited. This small cohort does not allow for robust conclusions regarding the diagnostic or prognostic value of circulating miR-7111-3p and APP. Therefore, the observed differential expression in serum should be considered a preliminary exploratory finding, and validation in large, well-powered clinical cohorts is required in future studies. Second, although EV-derived miR-7111-3p was shown to regulate NP69 cell behavior, the observed increase in proliferation and migration cannot be interpreted as evidence of full malignant transformation. These results likely reflect phenotypic alterations in normal nasopharyngeal epithelial cells induced by NPC-derived EVs. Long-term in vitro transformation assays or in vivo tumorigenicity studies using immunodeficient mouse models are required to determine whether these changes progress to malignant transformation. In addition, experimental evidence regarding the functional role and mechanistic regulation of miR-7111-3p within EVs remains limited and warrants further investigation. Then, experiments related to miR-7111-3p in EVs are relatively few, and its deeper mechanisms require further exploration. Third, this study did not directly validate the downstream signaling pathways of APP. The proposed association between APP and the MAPK/ERK pathway in EMT regulation is based on previous literature and remains a hypothetical mechanism in the context of NPC. Pathway-specific functional experiments are required to confirm this signaling axis. Moreover, the in vivo experiments were performed using a subcutaneous xenograft model, which is suitable for evaluating tumor growth but does not fully recapitulate invasion or distant metastasis. Future studies employing orthotopic or metastatic models are needed to comprehensively assess the anti-metastatic effects of miR-7111-3p. Finally, the relatively small clinical sample size limits the translational strength of this study. Large-scale prospective clinical studies are warranted to further validate the diagnostic and prognostic potential of miR-7111-3p and APP.

## Conclusion

This study systematically elucidates the tumor-suppressive role and underlying mechanism of miR-7111-3p in NPC. Our findings demonstrate that miR-7111-3p directly targets APP and suppresses its post-transcriptional expression, thereby inhibiting NPC cell proliferation, migration, invasion, and EMT. Notably, we provide the first evidence that EV-mediated delivery of miR-7111-3p plays a critical role in regulating the tumor microenvironment. NPC-derived EVs with reduced miR-7111-3p levels promote aberrant proliferative and migratory phenotypes in normal NP69 epithelial cells through APP upregulation. In vivo experiments further confirm that miR-7111-3p overexpression significantly suppresses tumor growth. Collectively, these findings not only deepen the understanding of the mechanisms underlying NPC malignant phenotypes and tumor growth, but also supply preliminary laboratory evidence for exploring potential molecular diagnostic markers and targeted therapeutic targets of NPC in follow-up large-cohort and preclinical investigations.

## Supplementary Information

Below is the link to the electronic supplementary material.

Supplementary Material 1

Supplementary Material 2

**Supplementary Material 3: Supplementary Figure S1**. miR-7111-3p regulates E-cadherin expression in NPC cells. (A, C, E, G) RT-qPCR analysis showing that miR-7111-3p overexpression significantly increases E-cadherin expression, while miR-7111-3p knockdown decreases E-cadherin levels in C666-1 and HK-1 cells. *n* = 3 biological replicates. (B, D, F, H) Western blot analysis confirming that E-cadherin protein expression is positively regulated by miR-7111-3p in NPC cell lines. *n* = 3 biological replicates.

**Supplementary Material 4: Supplementary Figure S2**. APP mediates miR-7111-3p-induced E-cadherin expression in NPC cells. (A, B) Western blot analysis showing that APP overexpression reverses miR-7111-3p-induced changes in E-cadherin in C666-1 and HK-1 cells. *n* = 3 biological replicates.

**Supplementary Material 5: Supplementary Figure S3**. Schematic diagrams of RNase protection assay and purified EV add-back treatment. (A) Schematic design of the RNase protection assay for isolated EVs. Purified EV suspensions were divided into three parallel groups: Control group treated with PBS only; Group 1 treated with RNase A alone; Group 2 co-treated with 0.1% Triton X-100 and RNase A. (B) RT-qPCR quantification of relative hsa-miR-7111-3p expression in EVs from the three experimental groups. *n* = 3 independent biological replicates. (C) Schematic workflow of purified EV add-back functional experiments in normal nasopharyngeal epithelial NP69 cells. NP69 cells were incubated with equal particle concentrations of three treatments: PBS blank control, EVs derived from Vector-transfected C666-1 NPC cells (EVs-Vector), or EVs derived from miR-7111-3p-overexpressing C666-1 NPC cells (EVs-miR-7111-3p OE).

**Supplementary Material 6: Supplementary Figure S4**. Molecular and phenotypic changes in NP69 cells after incubation with purified EVs carrying miR-7111-3p. (A) RT-qPCR analysis of intracellular hsa-miR-7111-3p expression in NP69 cells. n = 3 biological replicates. (B) RT-qPCR detection of APP mRNA expression in NP69 cells. n = 3 biological replicates. (C) Western blot analysis of APP protein levels in NP69 cells. (D) Colony formation assay assessing NP69 cell proliferative capacity. Representative colony staining images and quantitative counting data are shown. n = 3 biological replicates. (E) Transwell migtation assay detecting migration potential of NP69 cells. Representative crystal violet staining images. n = 3 biological replicates. (F) Wound healing assay evaluating NP69 migratory ability at 0 h and 24 h post-scratch creation. n = 3 biological replicates.

## Data Availability

Data and material will be made available on request.
