# Supplementary material for: EV-transferred miR-7111-3p inhibits malignant phenotypes and tumor growth in nasopharyngeal carcinoma via targeting APP-regulated epithelial-mesenchymal transition
Source: J Transl Med. 2026 Jul 30;24:1151. doi: 10.1186/s12967-026-08674-1 (PMC13551778; doi:10.1186/s12967-026-08674-1)
Supplement: Supplementary file 1 [file 12967_2026_8674_MOESM1_ESM.docx]

Table S1. Lentiviral sequence

| **Reagent** | **Sequence** | **Company** |
| --- | --- | --- |
| miR-7111-3p OE | upstream oligonucleotides: GGAAAGGACGAAACACCGGTTCCAAGTCTCTAAATATGCCAGTAAGAGCACCCACCAGG  downstream oligonucleotide: CGCCTAATGGATCCGAATTCAAAAAATGAGCAATGACACCAAGAAGTTGACAGCCAGGT | Genechem |
| miR-7111-3p sh | MicroRNA mature reverse complementary sequence: CTGGGAGGAGGGAAGAGAGGAT  ShRNA: AATTCAAAAAATCCTCTCTTCCCTCCTCCCAG | Genechem |
