# Supplementary material for: EV-transferred miR-7111-3p inhibits malignant phenotypes and tumor growth in nasopharyngeal carcinoma via targeting APP-regulated epithelial-mesenchymal transition
Source: J Transl Med. 2026 Jul 30;24:1151. doi: 10.1186/s12967-026-08674-1 (PMC13551778; doi:10.1186/s12967-026-08674-1)
Supplement: Supplementary file 2 [file 12967_2026_8674_MOESM2_ESM.docx]

Table S2. Antibody dilution ratio

| **Reagent** | **Dilution ratio** |  | **Company** |
| --- | --- | --- | --- |
| APP | 1:2000 |  | Yamaybio |
| N-cadherin | 1:1000 |  | CST |
| E-cadherin | 1:1000 |  | CST |
| Vimentin | 1:1000 |  | CST |
| Calnexin | 1:5000 |  | Proteintech |
| TSG101 | 1:1000 |  | Proteintech |
| HSP70 | 1:5000 |  | Proteintech |
| CD63 | 1:5000 |  | Proteintech |
| β-actin | 1:20000 |  | Proteintech |
